# LncRNA-SNHG6 promotes the progression of hepatocellular carcinoma by targeting miR-6509-5p and HIF1A

**DOI:** 10.1186/s12935-021-01835-w

**Published:** 2021-03-04

**Authors:** Xiaoxi Fan, Zhongwei Zhao, Jingjing Song, Dengke Zhang, Fazong Wu, Jianfei Tu, Min Xu, Jiansong Ji

**Affiliations:** 1 Key Laboratory of Imaging Diagnosis and Minimally Invasive Intervention Research, The Fifth Affiliated Hospital of Wenzhou Medical University/Affiliated Lishui Hospital of Zhejiang University/The Central Hospital of Zhejiang Lishui, Lishui, 323000 China; 2 Department of Radiology, The Fifth Affiliated Hospital of Wenzhou Medical University/Affiliated Lishui Hospital of Zhejiang University/The Central Hospital of Zhejiang Lishui, Lishui, 323000 China

**Keywords:** SNHG6, miR-6509-5p, HIF1A, Hepatocellular carcinoma, Proliferation, Migration, Invasion

## Abstract

**Background:**

Accumulating evidences have been reported that long noncoding RNAs play crucial roles in the progression of hepatocellular carcinoma (HCC). SnoRNA host gene 6 (SNHG6) is believed to be involved in several human cancers, but the specific molecular mechanism of SNHG6 in HCC is not well studied.

**Methods:**

In this study, we experimentally down-regulated the SNHG6 in two hepatocellular carcinoma cell lines in *vitro*, and then measured the proliferation, migration and invasion abilities and the apoptotic levels. Also, we performed the xenograft assay to investigate the function of SNHG6 during the tumor growth in vivo.

**Results:**

We found SNHG6 was highly expressed in HCC tissues. Next, using Hep3B and Huh7 cells, we confirmed knockdown of SNHG6 reduced the proliferation, migration and invasion abilities in *vitro*. Also, by bioinformatics analysis, further molecular and cellular experiments, we found miR-6509-5p bound to SNHG6 directly, and the expression level of HIF1A was regulated through SNHG6/miR-6509-5p axis. Finally, we found that down-regulation of SNHG6 dramatically reduced the tumor growth ability of Huh7 cells in *vivo*.

**Conclusions:**

We concluded that SNHG6/miR-6509-5p/HIF1A axis functioned in the progression of hepatocellular carcinoma, and could be the promising therapeutic targets during the development of hepatocellular carcinoma drugs.

## Background

Hepatocellular carcinoma (HCC) is one of the most common malignancies, accounting around 75% among all liver cancers [1]. The progression of HCC is contributed with several factors, involving hepatitis infection, environmental toxin, metabolic and genetic factors, which finally lead to cirrhosis and then HCC [2–7]. Increasing studies about the genetic factors have been reported, however, the pathophysiologic mechanisms involved in the progression of HCC are complicated and still unclear, which limits the therapeutic effects.

Large amount of evidences reveal that many genetic factors, especially the protein coding genes, contribute to the progression of HCC [8]. However, noncoding RNAs (ncRNAs), including long non-coding RNAs (lncRNAs) and small non-coding RNAs, are found to be involved in HCC progression in a competing endogenous RNA manner by directly binds to corresponding microRNAs [9–11]. Although lncRNAs are involved in diverse aspects in HCC progression, such as the proliferation, differentiation, and cell death, the identification of potential candidate lncRNAs remains at the early stage. SNHG6 (snoRNA host gene 6), a house keeping gene of the 5′ terminal oligopyrimidine family, is identified as a novel oncogene in diverse human cancers, including colorectal cancer, gastric cancer, ovarian clear cell carcinoma, human osteosarcoma and hepatocellular carcinoma [12–18]. Although many ncRNAs are identified as factors involved in HCC progression, the role of SNHG6 on the mechanisms of HCC initiation and progression still remains unknown.

Here, we found SNHG6 levels were remarkably increased in HCC tissues, especially in the aggressive cases of HCC. To further explore the role of SNHG6 on HCC progression using Hep3B and Huh7 cells, we found SNHG6 functioned on the proliferation, migration and invasion abilities of HCC. Then, we identified miR-6509-5p as the binding partner of SNHG6, and confirmed SNHG6 functioned as the sponge of miR-6509-5p. We further found the protein effector regulated by SNHG6/miR-6509-5p axis was HIF1A. Finally, by the tumor growth assay using nude mice, we confirmed down-regulation of SNHG6 significantly reduced the tumor size by decreasing the proliferation ability in HCC tissues. Taking together, we suggested that the SNHG6/miR-6509-5p/HIF1A axis functioned in the progression of HCC, and could be potential treatment target in HCC.

## Methods

### Ethics for HCC samples collecting and animal experiments

HCC samples from patients were collected under the guidelines of the Ethics committee. And the animal experiments were conducted under the guidelines of the Animal care and use committee. All nude mice were purchased from Shanghai SLAC Laboratory Animal Co., Ltd, and housed under the room temperature and a 12-h light–dark cycle.

### Total RNA extraction and quantitative PCR

Total RNA from human HCC samples and cell lines was extracted using Beyozol (R0011, Beyotime, Shanghai) according to the standard guideline. The extracted total RNA was reverse-transcripted to the complementary DNA using BeyoRT cDNA synthesis kit (D7168L, Beyotime, Shanghai). The expression levels of SNHG6 and HIF1A were quantified using qPCR SYBR Green Master Mix (DRR041A, Takara, China). GAPDH was selected as the internal reference gene.

### Culture and transfection of Hep3B and Huh7

Hep3B and Huh7 cells were purchased from ATCC and cultured in supplemented Minimum Essential Medium with Earle′s Balanced Salts (51415C, Sigma, America) supplemented with 10% FBS (fetal bovine serum) and 1% PS (penicillin and streptomycin). Cells were cultured at 37℃ using a humidified incubator containing 5% CO_2_ and 95% air. The small interfering RNA targeting SNHG6 and its corresponding scramble siRNA were synthesized by Genewiz, Suzhou, China. The sequences of siRNA for SNHG6 were as follows: si-SNHG6: AAAUGCUGCAUGCCACACUUGAGGU; scramble, UUCUCCGAACGUGUCACGUTT. The siRNA oligonucleotides were annealed and then cloned into pSuper vectors for the expression of siRNAs. Cell transfection assays were conducted using Lipofectamine 3000 (L3000075, ThemoFisher, America) according to the standard procedures.

### Immuno-histochemical staining

The cells were fixed with 4% paraformaldehyde (PFA) for 30 min at room temperature, washed with PBS for 3 times, and incubated with 0.5% Triton X-100 for 20 min. The proliferation status of the cells was then measured using BeyoClick™ *EdU* Cell Proliferation Kit (C0071, Beyotime, Shanghai). The tumor samples were collected, incubated in 4% PFA for fixation for 12 h, and sectioned into 20 μm slices. The slices were further incubated in 1% Triton X-100, primary antibody solution (Ki67, ab15580, Abcam, American) and secondary antibody solutions in sequence before imaging.

### Western blot

The lysate from Hep3B and Huh7 cells was prepared using RIPA lysis buffer (radio-immunoprecipitation assay buffer), and the protein concentration was measured with Pierce™ BCA Protein Assay Kit (23,225, ThermoFisher, American). The level of SOX2 was detected with the anti-SOX2 antibody (2748, Cell signaling, American). HIF1A antibody (AF1935, R&D Systems, America), cleaved caspase3 antibody (8202S, Cell Signaling Technology, America) and PCNA antibody (GTX100539, GeneTex, China) were used in this study.

### Migration and invasion assays

To assess the migration and invasion abilities of cells after down-regulation of SNHG6 and up-regulation of miR-6509-5p, 12 mm transwell with 3.0 µm pore polycarbonate membrane insert (3402, Corning, American) was employed. For the migration ability assessment, Hep3B and Huh7 cells were respectively seeded in the lower chamber at the density of 3 × 10^5^ cells. Cells were incubated till covering the entire bottom, scratched using the pipette tips, and then cultured for two days. For the invasion ability assessment, the chamber of the transwell insert was coated with Matrigel. Hep3B and Huh7 cells were respectively seeded in the chamber at the density of 3 × 10^5^ cells. Two days later, cells without migration were gently removed. Next, cells were fixed and then stained with 0.1% crystal violet. Finally, the wound-healing status of cells was imaged using the optical microscope.

### Luciferase assay

For the purpose of confirming the direct binding between SNHG6 and miR-6509-5p, SNHG6 fragment with the miR-6509-5p binding site and SNHG6 with mutated miR-6509-5p binding site were sub-cloned into the luciferase reporter vector. The SNHG-WT or SNHG6-MUT luciferase reporter vectors and miR-6509-5p were co-transfected into Hep3B cells. To confirm the direct binding of miR-6509-5p and 3′UTR of HIF1A mRNA, we constructed luciferase reporter vectors of miR-6509-5p using the previous strategy. The dual-luciferase reporter assay system (E1910, Promega, American) was employed to measure the luciferase activities under the guideline supplied by the manufacturer.

### Xenograft tumorigenesis

10 male BALB/C nude mice (6 weeks) were purchased from Shanghai SLAC Laboratory Animal Co.,Ltd, Shanghai, China and then randomly divided into two groups. To construct the SNHG6 stable down-regulated Huh7 cell line, Huh7 cells were infected with lentivirus containing si-SNHG6 and scramble sequences respectively. 5 × 10^6^ Huh7 cells stably expressing si-SNHG6 or its scramble siRNA were injected into the nude mice subcutaneously. 10 days after the tumor inoculation, the tumor tissues were dissected and analyzed.

### RNA immunoprecipitation assay

MiR-6509-5p mimics or miR-6509-5p-NC mimics was respectively transfected into Hep3B cells using Lipofectamine 3000, and the lysate of collected cells were prepared with centrifugation. To conduct the RNA immunoprecipitation assay, the anti-AGO antibody (MA5-23,515, ThemoFisher, American) was employed. And to confirm the direct binding of miR-6509-5p and SNHG6, the expression level of SNHG6 was measured using reverse transcription (RT) quantitative PCR.

### Distant metastasis assay

2 × 10^6^ Huh7 cells with stable down-regulated SNHG6 or 2 × 10^6^ control Huh7 cells were injected into nude mice through the tail vein injection. 6 weeks later, we dissected the lungs of these mice, and had the lungs fixed and stained for the counting of metastatic lung nodules.

### Statistical analysis

All statistical analyses were performed using GraphPad 8.0 under Students’ *t* test, and all data were presented as mean ± SEM from at least 3 independent experiments. Overall survival (OS) and progression-free survival (PFS) were presented using Kaplan–Meier curves. *P < 0.05, **P < 0.01, ***P < 0.001.

## Results

### SNHG6 contributed to the progression of hepatocellular carcinoma (HCC)

**Fig. 1:**
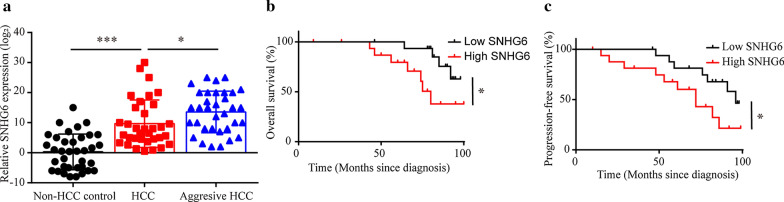
Increased expression level of SNHG6 was found in hepatocellular carcinoma (HCC) and led to poor prognosis. **a** The expression level of SNHG6 was increased in hepatocellular carcinoma tissues compared to normal controls, especially in aggressive HCC cases. **b** Overall survival (OS) and **c** progression-free survival (PFS) using Kaplan–Meier analysis between low and high SNHG6 expression patients with HCC. *P < 0.05, **P < 0.01, ***P < 0.001

We first explored the expression levels of SNHG6 in hepatocellular carcinoma (HCC) tissues, and found an increased SNHG6 level in HCC tissues compared to the non-HCC controls, especially in aggressive HCC cases (Fig. 1a). The expression level of SNHG6 and clinico-pathological characteristics of these 40 HCC patients were shown in Table 1. As SNHG6 level was positive correlated with HCC, we further conducted the analysis of overall survival (OS) and progression-free survival (PFS) based on Kaplan–Meier curves. The results shown that the expression level of SNHG6 was negatively correlated with abbreviated OS and PFS (Fig. 1b, c), which indicated SNHG6 contributed to the progression of HCC.

**Table 1:** The expression level of SNHG6 and clinic-pathological characteristics in 40 HCC patients

	SNHG6 Low	SNHG6 High	*P*-value
All	20	20	
Sex			0.744
Male	12	13	
Female	8	7	
Age			0.705
≤ 50	16	15	
> 50	4	5	
AFP(ng/ml)			0.197
≤ 20	14	10	
> 20	6	10	
Tumor size (cm)			0.337
≤ 5	10	13	
> 5	10	7	
Tumor numbers			0.212
Single	18	15	
Multiple	2	5	
Liver cirrhosis			0.705
Positive	15	16	
Negative	5	4	
Tumor nodes metastases stages			0.043
I	16	9	
II	4	8	
III	0	3	

### Down-regulation of SNHG6 inhibited the progression of HCC in vitro

**Fig. 2:**
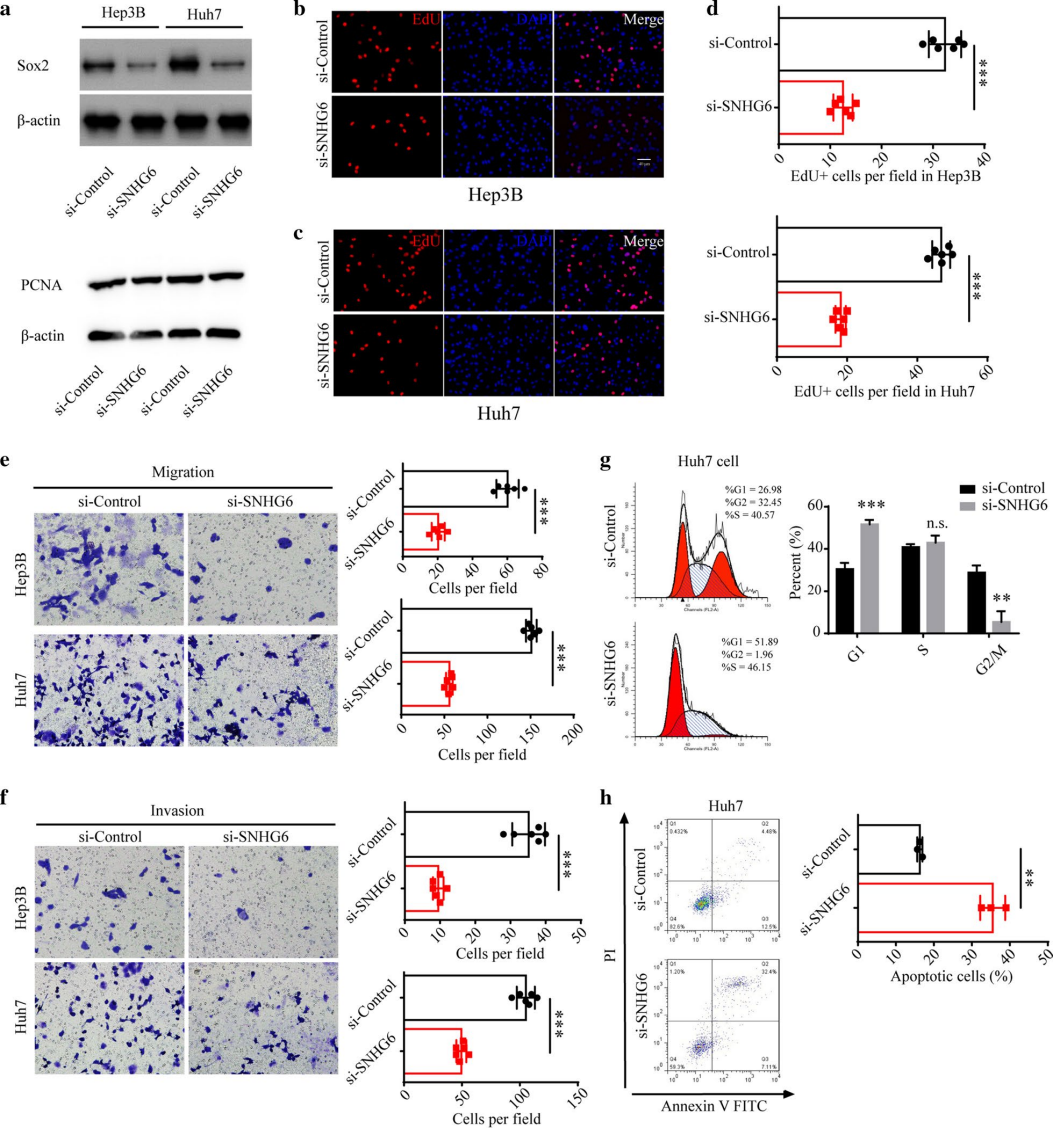
Down-regulation of SNHG6 inhibited the progression of HCC in *vitro*. **a** The expression levels of Sox2 and PCNA of Hep3B and Huh7 cells after down-regulation of SNHG6 were measured with western blotting. The proliferation ability of HCC cell lines, **b** Hep3B and **c** Huh7, was detected with EdU labeling. **d** EdU positive cells per field were calculated. **e** The migration and **f** invasion abilities of HCC cell lines after down-regulation of SNHG6 were determined with the transwell assay. **g** The percent of Huh7 cells in each cell cycles after SNHG6 knockdown. **h** The apoptosis level of Huh7 cells after SNHG6 knockdown. *P < 0.05, **P < 0.01, ***P < 0.001. Data were presented as mean ± SD from 6 independent experiments

We then investigated the role of SNHG6 in the progression of HCC using two HCC cell lines, Hep3B and Huh7. Firstly, we down-regulated SNHG6 using siRNAs in both cell lines, and found that SOX2 and PCNA protein levels were significantly decreased after knockdown of SNHG6, indicating the role of SNHG6 on the proliferation ability of HCC cells (Fig. 2a). We further confirmed that knocking down of SNHG6 led to decreased proliferation ability of Hep3B and Huh7 cells, using EdU labeling (Fig. 2b–d). Furthermore, we explored the role of SNHG6 on the migration and invasion abilities in HCC by conducting transwell assays. The results shown that knockdown of SNHG6 weakened the migration and invasion abilities of both Hep3B and Huh7 cells (Fig. 2e, f). To further examine the oncogenic potential of SNHG6, we analyzed the cell cycle parameters using FACS, and found SNHG6 knockdown led to cell cycle arrest at G1 phase (Fig. 2g). Additionally, we analyzed the cell apoptotic level using Annexin-V FACS, and found increased apoptotic cells after SNHG6 knockout (Fig. 2h). In summary, the in *vitro* data collected using Hep3B and Huh7 cells confirmed that SNHG6 indeed contributed to HCC by affecting the proliferation, migration and invasion abilities of HCC cells.

### SNHG6 functioned by directly binding to miR-6509-5p

**Fig. 3:**
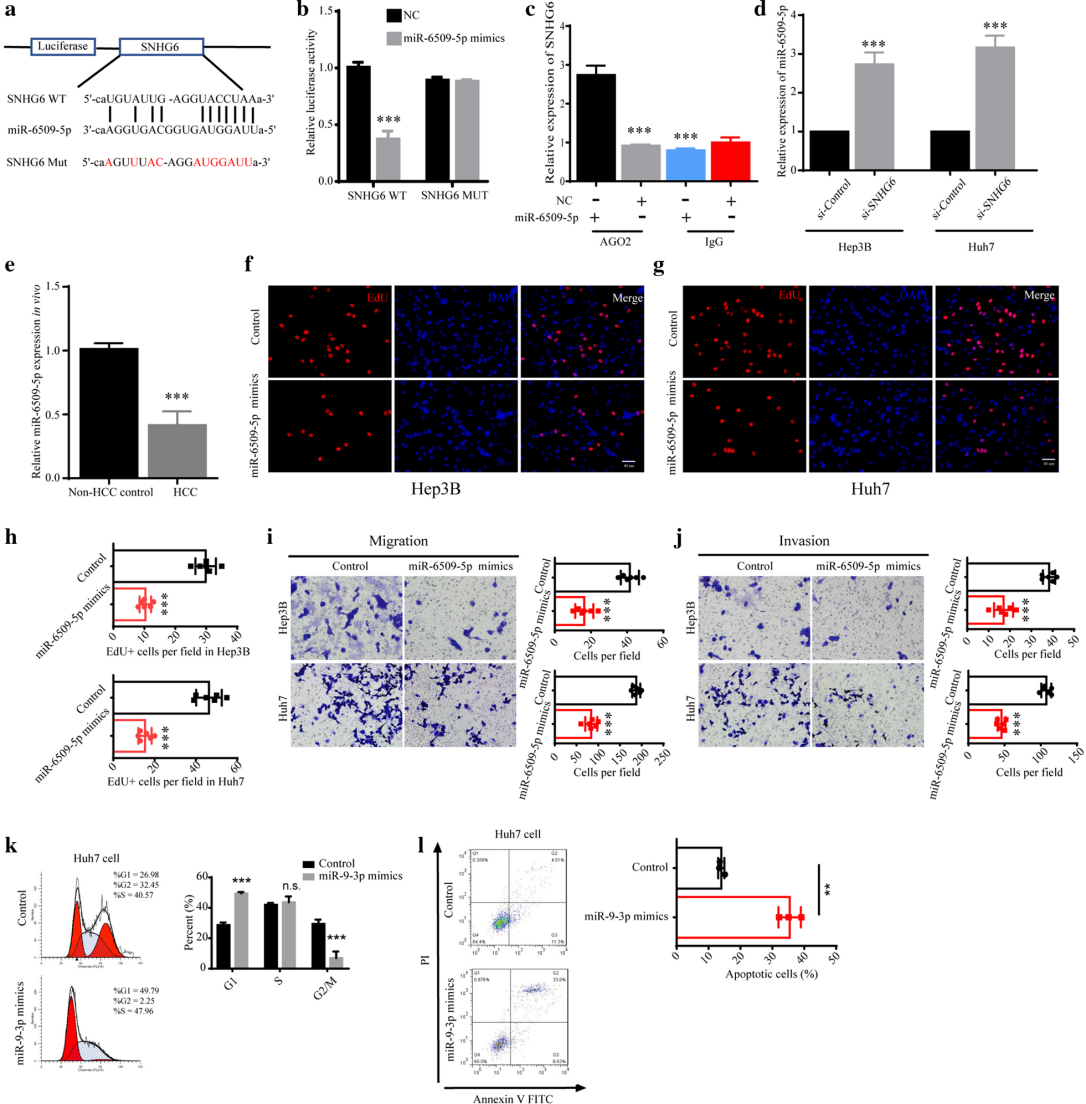
SNHG6 functioned as a sponge of miR-6509-5p. **a** The illustration of the luciferase assay to test the binding between SNHG6 and miR-6509-5p. **b** SNHG6-WT or SNHG6-MUT was co-transfected with miR-6509-5p, and the luciferase activities of SNHG6 reporter vectors were measured. **c** The binding between SNHG6 and miR-6509-5p was further confirmed using RNA immunoprecipitation (RIP) with the anti-AGO2 antibody. **d** The relative expression level of miR-6509-5p was detected using Hep3B and Huh7 cells. **e** The expression level of miR-6509-5p in HCC patients was measured. **f, g** The proliferation ability of HCC cell lines after up-regulation of miR-6509-5p with miR-6509-5p mimics was detected using EdU labeling, and **h** EdU positive cells per field were calculated. **i, j** The migration and invasion abilities of HCC cell lines after up-regulation of miR-6509-5p were evaluated using the transwell assay. **k** The percent of Huh7 cells in each cell cycles after the treatment of miR-6509-5p. **l** The apoptosis level of Huh7 cells after the treatment of miR-6509-5p. *P < 0.05, **P < 0.01, ***P < 0.001. Data were presented as mean ± SD from 6 independent experiments

Increasing evidences shown that lncRNAs played as molecular sponges to directly bind to miRNAs, and further affected the expression levels of the targeted proteins by degrading corresponding mRNAs [19–22]. For instance, lncRNA XIST could regulate the progression of hepatocellular carcinoma via targeting miR-497-5p to regulate the expression of PDCD4 [23]. Therefore, we conducted bioinformatics analysis and discovered miR-6509-5p as the potential binding partner of SNHG6. To test the directly binding of SNHG6 and miR-6509-5p, we constructed corresponding luciferase reporter vectors as shown (Fig. 3a). The significantly decreased luciferase activities of the SHNG6-WT and miR-6509-5p mimics group confirmed the binding effect (Fig. 3b). Furthermore, the directly binding effect between SNHG6 and miR-6509-5p in the AGO2-dependent manner was verified using the RNA immunoprecipitation assay (Fig. 3c). The expression level of miR-6509-5p was significantly increased after knockdown of SNHG6 (Fig. 3d). Also, the expression level of miR-6509-5p in HCC tissues was significantly higher compared to the health controls (Fig. 3e). To verify whether up-regulated miR-6509-5p mimicked the si-SNHG6 phenotype, we analyzed the role of miR-6509-5p on the progression of HCC. Results shown that similar to down-regulation of SNHG6, up-regulation of miR-6509-5p using miR-6509-5p mimics decreased the proliferation, migration and invasion abilities of Hep3B and Huh7 cells (Fig. 3f–j). To investigate the mechanism of the tumor suppressive role of miR-6509-5p underlying cell proliferation, we analyzed the cell cycle and cell apoptosis using FACS, and found that miR-6509-5p also led to cell cycle arrest at G1 phase and increased apoptosis level of Huh7 cells (Fig. 3k, l). We concluded that SNHG6 functioned through binding to miR-6509-5p.

### The expression of HIF1A was regulated by SNHG6/miR-6509-5p axis

**Fig. 4:**
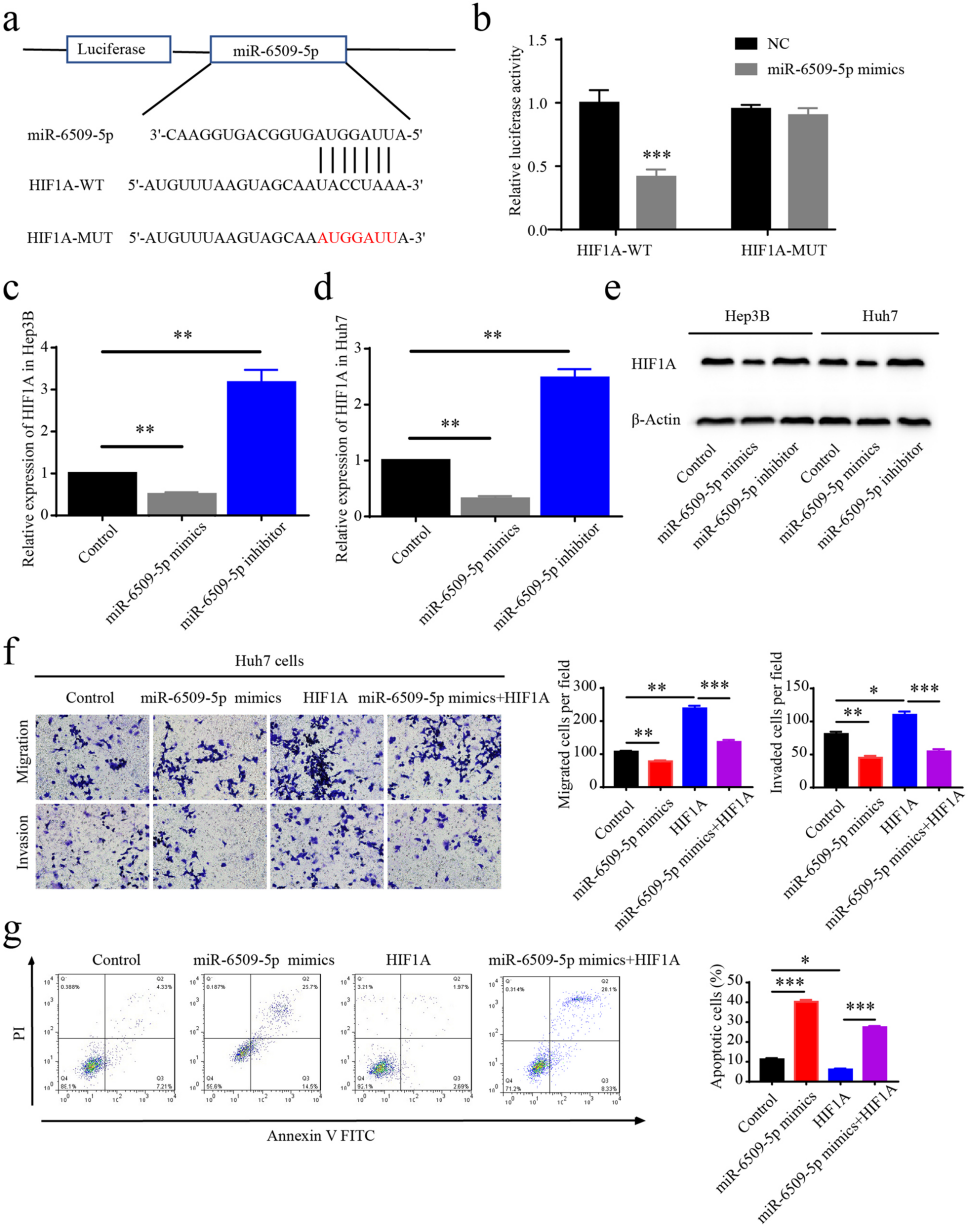
The expression of HIF1A was regulated by SNHG6/miR-6509-5p axis. **a** The illustration of the luciferase assay to test the binding between miR-6509-5p and HIF1A. **b** Luciferase activity of miR-6509-5p was measured to test the binding effect between miR-6509-5p and HIF1A. **c, d** The expression levels of HIF1A in Hep3B and Huh7 cells after treating with up- or down-regulation of miR-6509-5p were detected with quantitative PCR. **e** The levels of HIF1A in Hep3B and Huh7 cells after treating with up- or down-regulation of miR-6509-5p were detected with WB. **f** The migration and invasion abilities of Huh7 cells after up-regulation of HIF1A, and miR-6509-5p mimics reversed the effect. **g** The apoptosis level of Huh7 cells after up-regulation of HIF1A, and miR-6509-5p mimics reversed the effect. *P < 0.05, **P < 0.01, ***P < 0.001. Data were presented as mean ± SD from 3 independent experiments

Next, we screened the potential targets of miR-6509-5p related to cell cycle progression using TargetScan, and found that the 3′ UTR of HIF1A mRNA could directly bind to miR-6509-5p. HIF1A functioned in the proliferation of head and neck cancer cells by regulating the cell cycle progression [24]. Firstly, we constructed luciferase reporter vectors as illustration (Fig. 4a). The significantly reduced luciferase activities suggested the direct binding of miR-6509-5p and the 3′UTR of HIF1A mRNA (Fig. 4b). Secondly, we confirmed that the level of HIF1A mRNA was regulated by miR-6509-5p (Fig. 4c, d), and the level of HIF1A protein was also regulated by miR-6509-5p (Fig. 4e). Next, we investigated whether HIF1A was functionally related with miR-6509-5p by migration and invasion assays. The results shown that over-expression of HIF1A promoted the migration and invasion abilities of Huh7 cells, and miR-6509-5p mimics reversed these effects caused by HIF1A (Fig. 4f). At last, we tested the role of HIF1A on cell apoptosis, and found that up-regulation of HIF1A led to lower level of cell apoptosis, and miR-6509-5p mimics also functionally reversed these effect (Fig. 4g). In sum, we suggested that HIF1A was the protein effector regulated by SNHG6/miR-6509-5p axis.

### Down-regulation of SNHG6 inhibited the progression of HCC in vivo

**Fig. 5:**
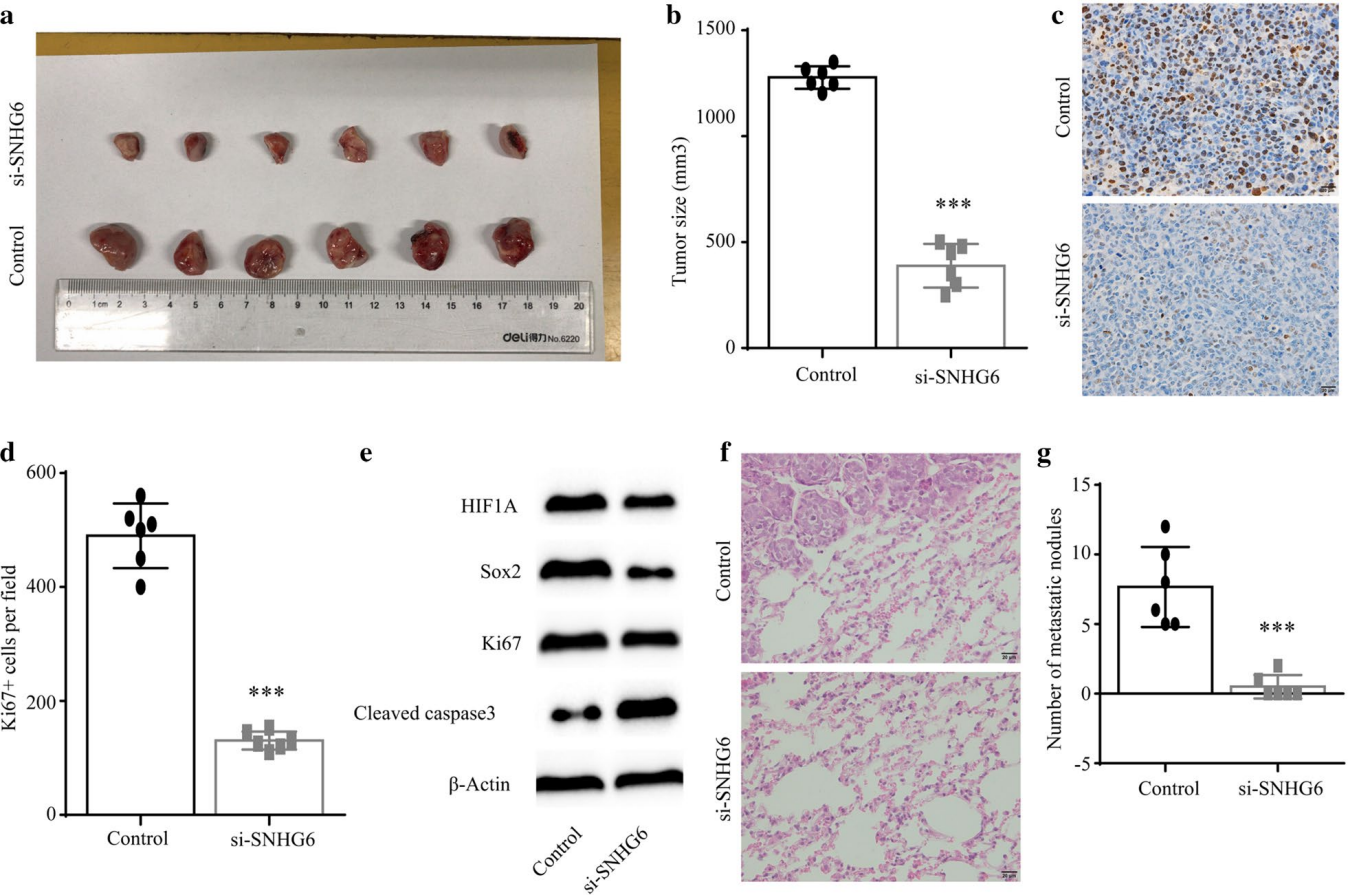
Down-regulation of SNHG6 suppressed the HCC growth in *vivo*. **a** The tumor growth ability of normal Huh7 cells and stable SNHG6 down-regulated Huh7 cells was evaluated using nude mice. **b** The tumor size was calculated. **c** The proliferated cells in the HCC tissues were labeled with Ki67 using immunohistochemistry methods. Ki67 positive cells were labeled brown. **d** Ki67 positive cells per field were calculated as the index of the proliferation status in the tumor tissues. **e** The levels of HIF1A, Sox2, Ki67 and cleaved caspase3 in the tumor tissues after SNHG6 knockdown. **f** Representative H&E staining images of the morphology of metastatic nodules in the lung of nude mice injected with Huh7 cells through the tail vein. **g** Quantification of the number of metastatic nodules in the lung. ***P < 0.001. Data were presented as mean ± SD from 6 independent experiments

After in *vitro* studies, we further explored the role of SNHG6 on the progression of HCC in *vivo*. We constructed stable down-regulated SNHG6 Huh7 cells using lentivirus containing si-SNHG6, and injected these cells into nude mice subcutaneously. 10 days after the inoculation, we dissected the subcutaneous xenograft tumors and measured the tumor size. Results shown that down-regulation of SNHG6 led to significantly decreased tumor volume (Fig. 5a, b). We further analyzed the tumor tissues by Ki67 labeling, and found the number of proliferating cells in the tumor tissues was significantly reduced (Fig. 5c, d, Additional file 1: Figure S1a). Next, we measured the expression level of HIF1A, and found that the expression of HIF1A was significantly reduced in si-SNHG6 tumor tissues (Fig. 5e). We then investigated the levels of proliferation and apoptosis markers, and found that after SNHG6 knockdown, the levels of proliferation markers, Sox2 and Ki67, were remarkably reduced, and the level of apoptosis was significantly increased (Fig. 5e). To investigate the effect of si-SNHG6 on the distant metastasis in *vivo*, we implanted the SNHG6 stable down-regulated Huh7 cells into nude mice by the tail vein injection. We found that comparing to the control group, down-regulation of SNHG6 significantly reduced the number of metastatic lung nodules, indicating si-SNHG6 inhibited the distant metastasis (Fig. 5f, g, Additional file 1: Figure S1b).

## Discussion

Hepatocellular carcinoma (HCC) remains to be a prevalent liver cancer for its poor prognosis and high recurrence rate [25]. HCC progression is contributed by diverse factors, especially genetic factors. Recently, including SNHG6, several long ncRNAs are identified as the potential targets responsible for HCC progression [18, 26–28]. However, the functional roles of SNHG6 in HCC progression remains unclear. In this study, we found the expression level of SNHG6 was positively correlated with HCC progression using human HCC tissues, and negatively correlated with shorter overall survival (OS) and progression-free survival (PFS) of HCC patients. Next, we further explored the role of SNHG6 on HCC progression on several aspects, including proliferation, migration and invasion abilities, and found knockdown of SNHG6 led to slower HCC progression. This finding indicated that the SNHG6 pathway could be a potential target for the treatment of HCC.

Long noncoding RNAs (lncRNAs) are noncoding RNAs which is longer than 200 nucleotides, and increasing data shown lncRNAs are involved in diverse cancers, including HCC [15, 18, 29–34]. LncRNAs typically function in a ceRNA manner. Therefore, we conducted bioinformatics analyses and identified miR-6509-5p as the potential binding target of SNHG6. The luciferase assay and RIP AGO assay confirmed the directly binding between SNHG6 and miR-6509-5p. In addition, we up-regulated miR-6509-5p in Hep3B and Huh7 cells, and found the cancer progression of cells treated with miR-6509-5p mimics was slowed down, indicating SNHG6 indeed functioned as the sponge of miR-6509-5p. The tumor suppressive role of miR-6509-5p may be employed during clinical therapeutics by delivering miR-6509-5p into the tumor tissues using adeno-associated virus (AAV).

Next, we conducted TargetScan analysis to investigate the potential protein targets of miR-6509-5p, and found the 3′UTR of hypoxia-inducible factor 1A (HIF1A) mRNA contained the binding sites for miR-6509-5p. HIF1A is known to regulate cellular adaption to hypoxic conditions and plays important roles in several cancers, including head and neck cancer, ovarian cancer and pancreatic cancer [24, 35, 36]. The remarkably decreased luciferase activities indicated the direct binding between miR-6509-5p and the 3′UTR of HIF1A mRNA. Also, the expression level of HIF1A was down-regulated by using miR-6509-5p mimics and up-regulated using miR-6509-5p inhibitor, confirming HIF1A was the target of miR-6509-5p. HIF1A is known to regulate cell adaptation to hypoxic conditions, and also plays important role in HCC, indicating that hypoxic conditions could be key factors during the progression of HCC. We should pay attention to the intrinsic characteristics of the genome mutations of HCC cells and the micro-environment of HCC tissues.

Finally, we performed the tumor growth assay using stable down-regulated SNHG6 Huh7 cells, and decreased tumor volume indicated knockdown of SNHG6 inhibited HCC progression. To explore the proliferation status of HCC tissues, we collected the tumor tissue and performed Ki67 labeling, and found the decreased proliferation in the HCC tissues treated with si-SNHG6. We confirmed that SNHG6 could regulated the proliferation and apoptosis of HCC cells in *vivo*, and suggested that the SNHG6/miR-6509-5p/HIF1A axis could serve as the potential target for alleviating the HCC progression.

To summarize, in this study, we discovered lncRNA-SNHG6 was highly expressed in HCC tissues and contributed to the proliferation, migration and invasion abilities of HCC. Furthermore, we identified the expression level of HIF1A was regulated through SNHG6/miR-6509-5p axis in a ceRNA manner. Finally, based on both in *vitro* and in *vivo* data, we could conclude that lncRNA-SNHG6 promotes HCC progression by miR-6509-5p/HIF1A axis, and provided novel potential targets for the development of HCC drugs.

## Conclusion

Our studies suggested that lncRNA-SNHG6 could promote the progression of hepatocellular carcinoma by targeting miR-6509-5p, and identified the cell cycle related protein, HIF1A, as the protein effector of the SNHG6/miR-6509-5p axis.

## Supplementary Information

**Additional file 1: Figure S1.** si-SNHG6 inhibited the proliferation and distant metastasis of HCC. a The proliferated cells in the HCC tissues were labeled with Ki67 using immunohistochemistry methods. Ki67 positive cells were labeled brown. b Representative H&E staining images of the morphology of metastatic nodules in the lung of nude mice injected with Huh7 cells through the tail vein.

## Data Availability

The data in this study for supporting the results are included within the article.
