# Supplementary figures and images for: LncRNA-SNHG6 promotes the progression of hepatocellular carcinoma by targeting miR-6509-5p and HIF1A
Source: Cancer Cell Int. 2021 Mar 4;21:150. doi: 10.1186/s12935-021-01835-w (PMC7931350; doi:10.1186/s12935-021-01835-w)

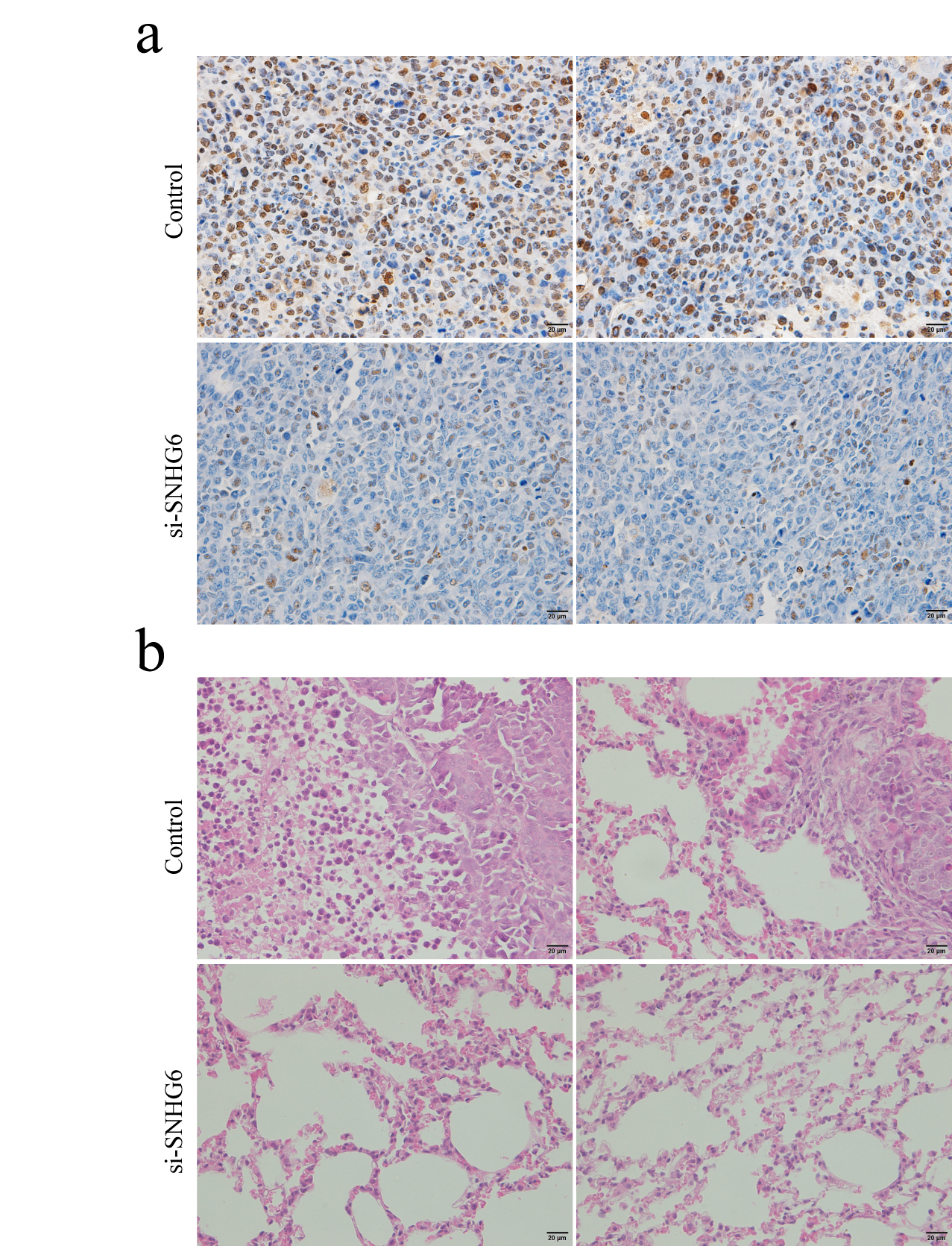

Supplement: Supplementary file 1 — Additional file 1: Figure S1. si-SNHG6 inhibited the proliferation and distant metastasis of HCC. a The proliferated cells in the HCC tissues were labeled with Ki67 using immunohistochemistry methods. Ki67 positive cells were labeled brown. b Representative H&E staining images of the morphology of metastatic nodules in the lung of nude mice injected with Huh7 cells through the tail vein. [file 12935_2021_1835_MOESM1_ESM.jpg]
